# Examining the correlates and drivers of human population distributions across low- and middle-income countries

**DOI:** 10.1098/rsif.2017.0401

**Published:** 2017-12-13

**Authors:** Jeremiah J. Nieves, Forrest R. Stevens, Andrea E. Gaughan, Catherine Linard, Alessandro Sorichetta, Graeme Hornby, Nirav N. Patel, Andrew J. Tatem

**Affiliations:** 1 Department of Geography and Geosciences, University of Louisville, Lutz Hall, Louisville, KY 40292, USA; 2 Department of Geography, Université de Namur, Rue de Bruxelles 61, 5000 Namur, Belgium; 3 Spatial Epidemiology Lab (SpELL), Université Libre de Bruxelles CP160/12, Avenue F.D. Roosevelt 50, 1050 Brussels, Belgium; 4 WorldPop, Geography and Environment, University of Southampton, Building 44, Room 54/2001, University Road, Southampton SO17 1BJ, UK; 5 Flowminder Foundation, Stockholm, Sweden; 6 GeoData, University of Southampton, Building 44, Room 44/2087, University Road, Southampton SO17 1BJ, UK; 7 Department of Geography and Geoinformation Science, George Mason University, 4400 University Drive, MS 6C3, Fairfax, VA 22030, USA

**Keywords:** population, mapping, census, dasymetric, disaggregation, random forests

## Abstract

Geographical factors have influenced the distributions and densities of global human population distributions for centuries. Climatic regimes have made some regions more habitable than others, harsh topography has discouraged human settlement, and transport links have encouraged population growth. A better understanding of these types of relationships enables both improved mapping of population distributions today and modelling of future scenarios. However, few comprehensive studies of the relationships between population spatial distributions and the range of drivers and correlates that exist have been undertaken at all, much less at high spatial resolutions, and particularly across the low- and middle-income countries. Here, we quantify the relative importance of multiple types of drivers and covariates in explaining observed population densities across 32 low- and middle-income countries over four continents using machine-learning approaches. We find that, while relationships between population densities and geographical factors show some variation between regions, they are generally remarkably consistent, pointing to universal drivers of human population distribution. Here, we find that a set of geographical features relating to the built environment, ecology and topography consistently explain the majority of variability in population distributions at fine spatial scales across the low- and middle-income regions of the world.

## 1. Introduction

While archaeologists have long stated that settlement patterns are complex and multi-factorial, geography has always been a determinant of the location of human settlements with humans primarily settling where resources are available, such as coastal areas and arable lands [1–5]. Access to sufficient resources to meet the needs of a population limit the population densities in any given location while other locations may have climates and topography that are less conducive to supporting human populations. However, the location of human populations is not simply determined by the natural environment, i.e. environmental determinism [6]. Since the agricultural revolution, humans have often been the drivers of change in the natural environment, modifying it in ways to better access resources/services (e.g. transportation networks, densification of services and production in urban areas) or to make the natural environment more productive and habitable (e.g. land conversion to agriculture, wetland drainage, irrigation, shelter in settlements) [7–11]. Sometimes humans have modified the environment in ways that make it less habitable, such as through pollution and desertification, or no longer habitable, such as in the cases of radiation in areas surrounding Chernobyl or desiccation of the Aral Sea [8,12,13]. With these changes, settlements and urban areas and populations continue to grow and their spatial distributions continue to evolve [14–16].

Between 2015 and 2050, the UN estimates that the global human population will grow by 2.4 billion [17]. Most of this projected change is anticipated to occur in the least developed countries and in urbanized areas [15,16]. Concurrently, Africa, Asia, Latin America and the Caribbean are estimated to experience the highest rates of urbanization [15]. As a part of this ‘urban transition’, the majority of Africa and Asia are experiencing large rates of internal migration, international migration and changes in the spatial distribution of natural population growth [15,16]. While Latin America and the Caribbean are predicted to experience decreasing urbanization rates, as was the trend through the 1990s and the early 2000s, the region is expected to have major demographic shifts. These rapidly changing magnitudes, composition and distribution of human populations imply a continued if not increasing need for high-resolution spatially explicit population maps that more accurately capture these changes to facilitate public health, sustainability and policy planning in general.

Over the past 20 years, the advancement of statistical techniques, availability of consistent geospatial data and rise in processing power have been leveraged to more accurately map populations over global scales. Such efforts include the simple gridding of census data matched to administrative boundaries that is undertaken for the Gridded Population of the World (GPW) project [18], and the use of satellite images of night-time lights to map urban areas and allocate populations to them, in the case of the Global Rural Urban Mapping Project (GRUMP) [19–21]. Other ongoing efforts, including LandScan [22–24], the Global Human Settlement Population Grid (GHS-POP) [25] and WorldPop [26], focus on a multivariate approach, utilizing multiple geospatial layers representing factors related to human population distributions to disaggregate areal unit-based census population counts to fine spatial resolution grid squares. These approaches can assess the contribution of different factors in explaining the observed population distributions (e.g. [26]), providing valuable data on the drivers and correlates of these patterns.

Despite the development of these multivariate approaches, there have been few globally representative comprehensive studies on the relationships between population densities, their associated covariates and the ancillary datasets that represent the covariates at a sub-national scale. Only basic within-country analyses have been undertaken in the course of validation or accuracy assessment, yet no analysis across low- and middle-income countries has occurred [26–29]. However, some local-scale case studies have investigated associations between covariates and population or residential land to better understand the correlates and drivers of population distributions in different settings [30,31]. Additionally, dasymetric modelling has evolved significantly over the past few years and provided important insights into the relationships between population and ancillary variables [32–35]. Such analyses have the potential to uncover fundamental patterns in the correlates and drivers of population distributions across the world.

Here, we undertake such an analysis for 32 low- and middle-income countries, focusing on answering the following two questions. (i) What datasets, representing drivers and associated landscapes of population distribution, are the most informative for accurately mapping populations at global scales?; (ii) What are the differences, in terms of relative importance of these datasets, between countries, between regions of countries and within regions of countries? By quantifying the relative importance of the drivers and correlates of human population distributions in relation to observed population densities, the question of how populations are distributed, and how this varies geographically, can begin to be addressed. Furthermore, it will allow informed development of new ancillary datasets with a high probability of importance when placed within a modelling framework and potentially lead to more informed covariate choices in population modelling that can expand the possible end-use applications of the population data. Moreover, by better depicting the relative importance of the drivers and associated landscapes of populations at the global and regional scales the accuracy and precision of high-resolution population mapping and construction of future scenarios will be furthered, benefitting all down-stream applications.

## 2. Material and methods

To assess the relationships between population densities and candidate correlates and drivers, we built a machine-learning-based modelling framework to expose the relationships between sub-national boundary-matched population census data and a library of geospatial datasets. The population models considered in this study are based on the random forest (RF)-based method as described in Stevens *et al*. [26]. We took the RF regression model objects for each sample country which were trained at the administrative unit level of the corresponding census-based population data, extracted the covariate importance metrics, standardized what the covariates were representing to facilitate comparisons across models and analysed these data for differences between and within covariate classes as well as within each covariate class between all countries, between regions and within regions to begin to address the possibility of geographic variability in these relationships.

### 2.1. Random forest-based population models

RFs are a non-parametric, nonlinear statistical method that falls within a category of machine-learning methods known as ‘ensemble methods’. Ensemble methods take individual decision trees that are considered ‘weak learners’ and combine them to create a ‘strong learner’. The benefits of ensemble methods are that generalizability is increased, performance on large or small datasets is improved and the ability of the method to model difficult learning tasks is more effective. Compared with other ensemble methods RFs are robust to noise, small sample sizes and over-fitting, yet they need little in the way of parameter specifications [36–39].

RFs independently generate *k* number of unpruned decision trees using ‘bagging’ [37,40]. Once a decision tree is grown, the one-third of the bagged training data that the tree was not grown upon remain and are known as the ‘out-of-bag’ (OOB) data. The decision tree applied to these data and the accuracy of the tree, as measured by the mean squared error (m.s.e.), are stored as the OOB error for that tree [37]. The prediction error of the entire RF model can be estimated by averaging the OOB error of all trees [37]. The OOB error is also used for estimating covariate importance by randomly permutating a given covariate's OOB data with random noise and calculating the average per cent increase in the mean squared error, hereafter the Per.Inc.m.s.e., across all trees of the RF model which used the covariate [37]. For more details on the construction of RFs, see Breiman [37] and Liaw & Wiener [38].

**Figure 1.**
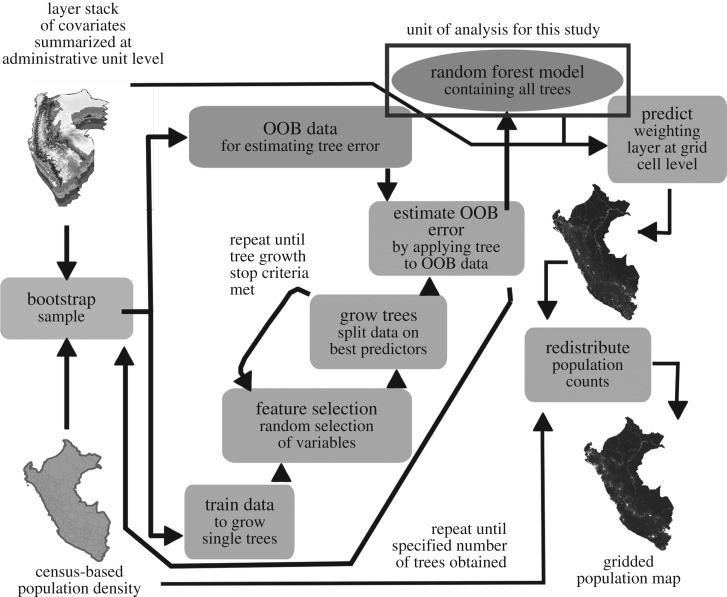
General process of using a random forest to created gridded population maps following Stevens *et al*. [26], where ‘out-of-bag’ (OOB) data are the approximately one-third of the data not sampled for training any single tree.

The RF method outlined by Stevens *et al*. [26] uses an RF regression model and dasymetric mapping methods in a three-step process to estimate a population layer from input census and covariate data. The general steps are as follows: (i) iterative covariate selection for the RF model, (ii) the fitting of the RF model, using all available census units, and creation of a population density weighting layer from the created RF model, and (iii) the dasymetric redistribution of population counts from census-based administrative units to grid cells [29] using the population density weighting layer [26,32,33]. We give a general schematic of the RF process described by Stevens *et al*. [26] in figure 1. The covariate selection process is identical to step 2, but iterates until the removal of all covariates with a Per.Inc.m.s.e. less than zero. Data input to an RF model varies on a country-by-country basis with high-resolution country-specific datasets being used over coarser resolution default datasets, when available. This last detail required the standardization of what each covariate more generally represented to facilitate comparison across models.

### 2.2. Census data

**Figure 2.**
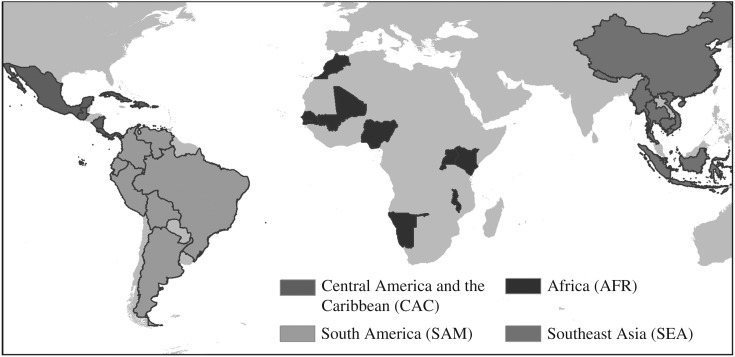
Countries for which boundary-matched census data were used in this study, from Africa, Central America and the Caribbean, South America and Southeast Asia.

For this investigation, we sampled countries (*n* = 32) from low- and middle-income countries in four regions of the world where available boundary-matched census data were available at an average spatial resolution (ASR) of 100 km^2^ or below: Africa, Central America and the Caribbean (C. America and the Caribbean), South America (S. America) and Southeast Asia (S.E. Asia) [41]. The sampled countries, shown in figure 2, were modelled upon census data from varying years, with differing ASRs [41] of administrative units, and people per administrative unit, shown in table 1. These regions were selected because of their continued and rapidly growing importance in relation to world population [15,17].

**Table 1.** Sampled countries and selected characteristics including the variance explained by the country-specific random forest model. admin., administrative; avg., average.

country	ISO	region	census year (admin. level)	admin. units	avg. spatial resolution (km^2^)	people per unit (thousands)	variance explained
Kenya	KEN	Africa	1999 (5)	6606	9	4.3	83%
Morocco	MAR	Africa	2004 (4)	1497	16	21	80%
Mali	MLI	Africa	2009 (4)	687	43	22	85%
Malawi	MWI	Africa	2008 (2)	12 557	22	59	79%
Namibia	NAM	Africa	2011 (2)	5475	12.28	21	96%
Nigeria	NGA	Africa	2006 (2)	774	34	205	88%
Rwanda	RWA	Africa	2002 (4)	9183	1.68	1.2	69%
Senegal	SEN	Africa	2009 (4)	331	24	37	91%
Uganda	UGA	Africa	2002 (4)	5018	7	6	85%
Bolivia	BOL	C. America and Caribbean	2012 (2)	112	97.7	91	65%
Costa Rica	CRI	C. America and Caribbean	2011 (3)	469	10.4	9.8	92%
Cuba	CUB	C. America and Caribbean	2012 (2)	168	25.6	68	82%
Dominican Republic	DOM	C. America and Caribbean	2010 (3)	155	17.6	64	86%
Guatemala	GTM	C. America and Caribbean	2012 (2)	333	18.0	46	80%
Haiti	HTI	C. America and Caribbean	2009 (4)	570	6.9	17	84%
Mexico	MEX	C. America and Caribbean	2010 (2)	2456	28.0	48	92%
Nicaragua	NIC	C. America and Caribbean	2012 (3)	137	29.4	43	79%
Panama	PAN	C. America and Caribbean	2010 (2)	74	31.04	49	74%
Puerto Rico	PRI	C. America and Caribbean	2010 (1)	78	13.3	48	74%
Argentina	ARG	S. America	2010 (2)	526	73.0	78	88%
Brazil	BRA	S. America	2010 (4)	5565	5.1	36	84%
Colombia	COL	S. America	2013 (4)	1115	32.0	42	84%
Ecuador	ECU	S. America	2010 (4)	978	16.2	15	82%
Peru	PER	S. America	2012 (2)	194	81.7	155	63%
Venezuela	VEN	S. America	2011 (2)	339	51.6	87	71%
Cambodia	KHM	S.E. Asia	2008 (3)	1621	10.51	8.6	92%
China	CHN	S.E. Asia	2010 (4)	2922	57.28	458	95%
Indonesia	IND	S.E. Asia	2010 (4)	79 277	4.91	3.0	81%
Myanmar	MMR	S.E. Asia	2014 (3)	326	45.29	164	94%
Nepal	NEP	S.E. Asia	2011 (4)	3973	6.08	6.8	92%
Thailand	THA	S.E. Asia	2010 (3)	7416	23.67	9.0	88%
Vietnam	VNM	S.E. Asia	2010 (3)	688	21.85	123	93%

### 2.3. Geospatial covariates and standardization

Human population density is highly correlated with environmental and physical factors [35], which can influence distributions of population. As indicated by the literature and availability of global data, the following factors were identified and used as predictive covariates: intensity of night-time lights [42], energy productivity of plants [43], topographic elevation and slope [44,45], climatic factors [46], type of land cover (LC) [27] and presence/absence of roads [47], water features [48], human settlements and urban areas [49], protected areas [50] and locations of points of interest (POIs) and facilities such as health centres and schools [51]. Rather than attempt to standardize the input covariates between countries, we used the most contemporary available datasets on a country-by-country basis to produce the population maps. See Stevens and co-workers [26] and [29] for a typical set of ancillary data included in a given model, with further details provided in Lloyd *et al*. [52].

For every model run, information about the RF model settings, covariates and their importance, metadata on the covariate datasets themselves and the general results of the RF model were output to summary files, which are included in the electronic supplementary material. From those summaries, we extracted the region modelled, the total variance explained by the model, the covariate names and the Per.Inc.m.s.e. for every covariate included in the model [37]. We then examined the covariates for all sampled countries to reclassify them into the covariate classification groups shown in table 2 as informed by common themes through the literature and patterns seen through population modelling of numerous countries. The primary purpose of this classification system was to facilitate comparisons between the country models via a standardized framework.

**Table 2.** Reclassification scheme to standardize covariates into variable classes representing spatial drivers and determinants of population. LC, thematically classified land cover; LU, classified land use; nat., natural; OSM, Open Street Map; semi.-nat., semi-natural; veg., vegetation. Note: The references are not exhaustive, but are characteristic of most models. Any of these covariates could be replaced by a country-specific dataset sourced from a one-off source or country partner. Refer to country-specific metadata files provided with the source download from www.worldpop.org.

aggregated variable class	drivers, correlates and covariates
natural/semi-natural vegetation land cover	LC nat. and semi-nat. veg.—woody [53,54]
	LC nat. and semi-nat. veg.—shrubs [53,54]
	LC nat. and semi-nat. veg.—herbaceous [53,54]
	LC nat. and semi-nat. veg.—other mix [53,54]
	LC nat. and semi-nat. veg.—aquatic veg. [53,54]
cultivated/managed land cover	LC cultivated terrestrial and managed lands [53,54]
natural bare surfaces land cover	LC natural bare surface [53,54]
artificial surface land cover	LC urban areas [53,54]
	LC rural settlement [53,54]
no data	LC no data [53,54]
residential land use	LU residential [55]
non-residential land use	LU industrial [55]
	LU farms [55]
protected land use	e.g. protected natural areas [56]
general classified land use	e.g. multiple classified land uses provided to model as a single covariate [55]
urban/suburban extents	global human settlement layer [57]
	Schneider MODIS [58]
built environment and urban/suburban proxies	LC urban areas+LC rural settlement [53]
	lights at night imagery [59]
	building footprints [55]
classified populated place (hierarchical)	e.g. city, town, village, etc. [55]
transportation networks	roads [55,60]
	railways [55]
climatic/environmental	elevation and slope [61]
	net primary productivity [62]
	temperature [63]
	precipitation [63]
facilities and services	schools [55]
	police [55]
	nutrition [55]
	health facilities [55]
places and POIs	OSM places [55]
	OSM POIs [55]
rivers/waterbodies/waterways	LC water [53,54]
	rivers [55]
	waterbodies/waterways [55,60]
populated place	e.g. gazetteer-type data [55,60]

We would expect that covariates within the urban/suburban extents and built environment and urban/suburban proxy classes would be the most important for predicting population density as these typically capture settlements either implicitly or explicitly [10,64–66]. Transportation networks and facilities and service classes would also be expected to be consistently important as transportation networks exist solely to facilitate the movement of people, goods and ideas [66]. Responding to the classic ‘location–allocation’ problem, facilities and services, e.g. schools and health centres, are often located to promote access by and service to a population. Rivers/waterbodies/waterways are unique in that they can be used by people as both a transportation network and a resource, an attraction for population, but, in some cases, could be perceived as more hazard than resource, e.g. floods, and would therefore serve as a disincentive for a population locating near them. Previous studies have shown that landcover classes can be used for predicting population density by predicting either their absence, e.g. natural or bare surface land cover, or their presence due to their direct impact on land use (LU), e.g. cultivated land cover [8,27,32].

### 2.4. Analysis

From the independently modelled countries, we synthesized generalized data on the relative importance of various covariates in predicting population densities. All analysis and data handling was performed in the R Statistical Environment, version 3.2.2, with *α* = 0.05 significance levels and appropriate corrections for multiple outcomes where indicated [67].

To account for the differing number of total covariates in each country's model, we calculated a weighted importance rank (WIR). Within each country, we ranked covariates by descending Per.Inc.m.s.e. and then weighted them by the total number of covariates in the final model for a given country, calculated as
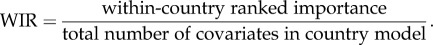


Within a given country, a WIR of zero indicates the covariate of highest importance and a WIR of 1 is the least important covariate. Hereafter, unless explicitly stated, within the text, variable class importance is referring to the WIR. To examine potential differences in variable class importance, we used both analytical and graphical methods.

Given the non-normal nature of the covariate importance data, we used the non-parametric form of the Kruskal–Wallis test to test for significant differences between covariate classes across all countries [68]. The inter-regional analyses were of a hierarchical nature using data subsets of a given covariate class and using the region category as the grouping variables, but still using the Kruskal–Wallis test [68,69]. The intra-regional analyses subset the data to a given region and a given covariate class then used a Kruskal–Wallis test to determine whether significant differences in importance for the given covariate class existed between countries of the same region [68]. If any of the Kruskal–Wallis tests were significant they were followed up with *post hoc* Dunn tests, using Holm's correction for multiple outcomes, to determine between which covariate classes or regions the significant differences occurred [70,71].

## 3. Results

The consistent patterns of covariate importance to predicting population density were observed between all sampled countries globally, with similar patterns observed between regions of countries. The correlates pertaining to urban areas and, more surprisingly, topographical features were the most important predictors of population density at all scales of analysis and were the only covariate categories which were consistently significantly more important than other categories, again at all scales.

### 3.1. Global

**Figure 3.**
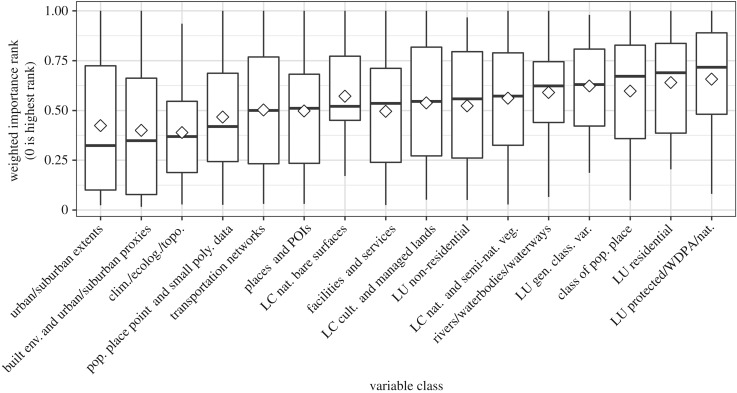
Global variable class weighted rank of importance based upon covariates included in a given country's final model, where zero represents the highest rank. The mean is represented by a white diamond; the median is represented by the black bar; and the whiskers represent the maximum and minimum values within 1.5× the inter-quartile range. See table 2 for descriptions and references for the variable classes. LC, land cover; LU, land use; WDPA, World Database on Protected Areas.

We present global covariate importances in figure 3. The five most important covariate classes, in descending order of median importance, were urban/suburban extents (0.32), built environment and urban/suburban proxies (0.35), climatic/environmental variables (0.37), populated place covariates (0.42) and transportation networks (0.50). This result matches expectations, as the five most important covariate classes (figure 3) are also the most often included in the final population models.

Globally, for predicting population density, we found that built environment covariates were significantly more important than classified populated place (*p* < 0.01), natural/semi-natural vegetation LC (*p* < 0.01), general classified LU (*p* = 0.04), protected LU (*p* < 0.01) and rivers/waterbodies/waterways covariates (*p* < 0.01). We also found that urban/suburban extents were significantly more important than protected LU (*p* < 0.01). Furthermore, we observed that climatic/environmental variables were significantly more important than populated place (*p* < 0.01), natural/semi-natural vegetation LC (*p* < 0.01), general classified LU (*p* = 0.02), protected LU (*p* < 0.01) and rivers/waterbodies/waterways covariates (*p* < 0.01). Interestingly, we observed no significant difference in importance between the urban/suburban extents and the built environment and urban/suburban proxy classes. In table 3, we show test results for significant differences between covariates of the top five important covariate classes when compared with all other covariate classes. The complete results are detailed in the electronic supplementary material.

**Table 3.** Selected results of the pairwise *post hoc* Dunn test with Holm's correction for multiple outcomes of global WIR of covariate classes. See table 2 for descriptions and references for the variable classes. LC, land cover; LU, land use. See the electronic supplementary material for results across all classes. Global Kruskal–Wallis results: d.f. = 15, chi-squared = 96.147, *p* < 0.01. Full precision of the values is provided in the electronic supplementary material.

variable class	corrected *Z*-value (corrected *p*-values)*				
	built env. and urban/suburb. proxies	climatic/environmental	populated place	transportation networks	urban/suburb. extents
class of pop. place	5.04 (<0.01)	5.53 (<0.01)	2.41 (1.00)	2.41 (1.00)	3.43 (0.06)
climatic/environmental	0.30 (1.00)	—	1.49 (1.00)	3.20 (0.14)	0.72 (1.00)
facilities and services	2.06 (1.00)	2.36 (1.00)	0.48 (1.00)	0.16 (1.00)	1.27 (1.00)
cultivated/managed LC	3.43 (0.37)	3.20 (0.14)	1.18 (1.00)	0.74 (1.00)	1.98 (1.00)
natural/semi-natural vegetation LC	4.82 (<0.01)	5.44 (<0.01)	1.90 (1.00)	1.76 (1.00)	2.98 (0.28)
nat. bare surfaces LC	3.19 (0.14)	3.46 (0.06)	1.60 (1.00)	1.27 (1.00)	2.35 (1.00)
general classified LU	3.58 (0.04)	3.81 (0.02)	2.15 (1.00)	1.93 (1.00)	2.84 (0.42)
non-residential LU	1.55 (1.00)	1.71 (1.00)	0.64 (1.00)	0.25 (1.00)	1.16 (1.00)
protected LU	5.52 (<0.01)	5.91 (<0.01)	3.19 (0.14)	3.31 (0.10)	4.13 (<0.01)
residential LU	3.37 (0.08)	3.56 (0.04)	2.16 (1.00)	1.93 (1.00)	2.77 (0.52)
places and POIs	2.08 (1.00)	2.38 (1.00)	0.51 (1.00)	0.11 (1.00)	1.29 (1.00)
populated place	1.26 (1.00)	1.49 (1.00)	—	0.68 (1.00)	0.69 (1.00)
rivers/waterbodies/waterways	4.80 (<0.01)	5.28 (<0.01)	2.27 (1.00)	2.20 (1.00)	3.27 (0.11)
transportation networks	2.76 (0.52)	3.20 (0.14)	0.68 (1.00)	—	1.61 (1.00)
urban/suburban extents	0.48 (1.00)	0.72 (1.00)	0.69 (1.00)	1.61 (1.00)	—

### 3.2. Inter-regional

Another POI was that the strong patterns of association seen at the global level were largely consistent when drivers and correlates were examined between regions. The only significant differences between regions were seen for the non-residential LU variable and the rivers/waterways/waterbodies variable, the latter shown in table 4. Non-residential LU was significantly more important in C. America and the Caribbean than in S. America (*p* = 0.02; *Z* = 2.35). As shown in table 4, rivers/waterbodies/waterways were significantly more important in Africa (*p* < 0.01; Z = 3.78) and S.E. Asia (*p* < 0.01; Z = 4.08) than in C. America and the Caribbean.

**Table 4.** Results of the pairwise Dunn test with Holm's correction for differences in WIR of variable class by region within the rivers/waterbodies/waterways class. Corrected *Z*-score and corrected *p*-value, in parentheses, are given. Full results for all variable classes between regions, including non-significant findings, are provided in the electronic supplementary material. Kruskal–Wallis results: d.f. = 3, chi-squared = 20.281, *p* < 0.01.

region	Africa	C. America and Caribbean	S. America
C. America and Caribbean	3.78 (<0.01)	—	—
S. America	1.21 (0.45)	2.32 (0.08)	—
S.E. Asia	0.77 (0.45)	4.08 (<0.01)	1.79 (0.22)

**Figure 4.**
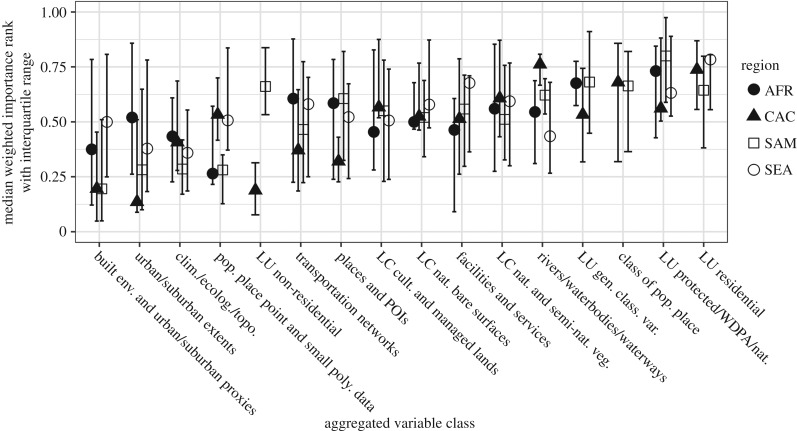
Regional line and dot plot of variable class WIR with the median marked by the dot and the inter-quartile range demarcated by brackets. Note that not all regions have all variable classes. See table 2 for descriptions and references for the variable classes.

The consistency of importances within covariate classes across regions becomes apparent when plotting the importance, with the inter-quartile range (IQR), as done in figure 4. It can first be noted that many of the covariate class IQRs overlap between regions, with very similar median importances and variation seen for climatic/environmental covariates, transportation networks and cultivated/managed LC. There is more variation in importance than expected between regions for covariates of urban/suburban extents and the built environment and urban/suburban proxies. The findings from table 4, and all the inter-regional tests included in the electronic supplementary material, agree with the distributions shown in figure 4.

### 3.3. Intra-regional

**Figure 5.**
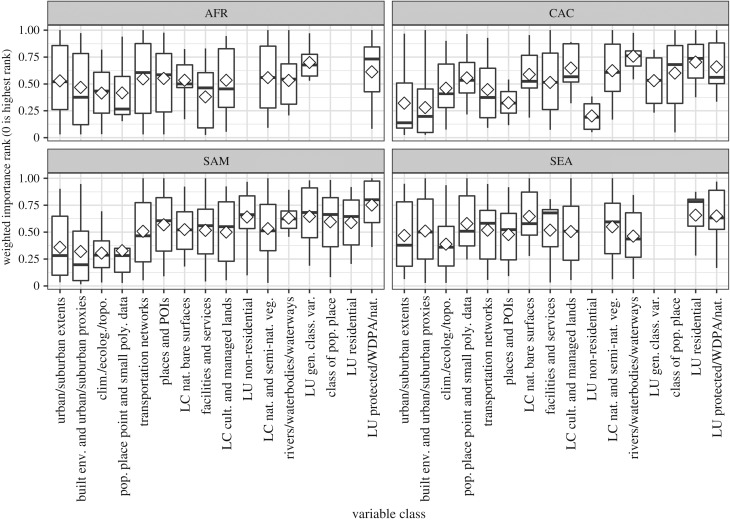
Regional variable class weighted rank of importance based upon covariates included in a given country's final model, where zero represents the highest rank. The mean is represented by a white diamond; the median is represented by the black bar; and the whiskers represent the maximum and minimum values within 1.5× the inter-quartile range. See table 2 for descriptions and references for the variable classes.

Like global patterns, there were no differences between the importance of the covariates urban/suburban extents and built environment and urban/suburban proxies within any region. Within any single region, we found no significant differences in patterns of importance between countries for all given covariate classes. However, between covariate classes across all countries within a given region, we found significant differences within the C. America and the Caribbean and S. America regions and display these in table 5. Similar to the global results, we found within S. America that built environment and urban/suburban proxies were significantly more important than classified populated place (*p* < 0.01), protected LU (*p* < 0.01) and rivers/waterbodies/waterways covariates (*p* = 0.01). Also within S. America, we found that climatic/environmental variables were significantly more important than classified populated place (*p* < 0.01), natural/semi-natural vegetation LC (*p* = 0.02), general classified LU (*p* = 0.04), protected LU (*p* < 0.01) and rivers/waterbodies/waterways covariates (*p* < 0.01). For C. America and the Caribbean, we found that the covariates regarding built environment and urban/suburban proxies (*p* < 0.01), transportation networks (*p* = 0.03), urban/suburban extents (*p* < 0.01) and climatic/environmental variables (*p* = 0.02) were significantly more important than rivers/waterbodies/waterways covariates. Additionally, built environment and urban/suburban proxies were found to be significantly more important than classified populated place (*p* = 0.01), natural/semi-natural vegetation LC (*p* < 0.01) and protected LU (*p* < 0.05). Full results including the non-significant findings are included in the electronic supplementary material. We illustrate the consistency of the importance of distribution and their relative importance regionally for each covariate class graphically in figure 5.

**Table 5.** Selected results of the pairwise Dunn test with Holm's correction for differences in WIR by region between variable classes. Corrected *Z*-scores and corrected *p*-values, in parentheses, are given. Full results between all variable classes within regions, including non-significant findings, are provided in the electronic supplementary material. Full precisions of values are provided in the electronic supplementary material.

region	variable class	built env. and urban/suburban proxies	climatic/environmental	urban/suburban extents	transportation networks	populated place
S. America	classified populated place	4.54 (<0.01)	5.09 (<0.01)	2.73 (0.63)	1.69 (1.00)	2.76 (0.57)
	natural/semi-natural vegetation LC	3.73 (0.10)	3.73 (0.02)	1.94 (1.00)	0.46 (1.00)	2.06 (1.00)
	general classified LU	3.34 (0.09)	3.56 (0.04)	2.48 (1.00)	1.50 (1.00)	2.57 (0.95)
	protected LU	4.29 (<0.01)	4.52 (<0.01)	3.32 (0.10)	2.55 (1.00)	3.36 (0.08)
	rivers/waterbodies/waterways	3.82 (0.01)	4.14 (<0.01)	2.65 (0.77)	1.63 (1.00)	2.72 (0.63)
C. America and Caribbean	classified populated place	3.85 (0.01)	1.76 (1.00)	2.63 (0.88)	1.84 (1.00)	0.39 (1.00)
	natural/semi-natural vegetation LC	4.62 (<0.01)	2.30 (1.00)	3.03 (0.26)	2.36 (1.00)	0.61 (1.00)
	protected LU	3.52 (<0.05)	1.88 (1.00)	2.66 (0.81)	1.95 (1.00)	0.75 (1.00)
	rivers/waterbodies/waterways	5.66 (<0.01)	3.66 (0.03)	4.07 (<0.01)	3.69 (0.03)	1.75 (1.00)

## 4. Discussion

The majority of predicted population growth across the globe by 2050 is expected to occur in low- and middle-income countries [14,15,17]. With this predicted growth in population and urbanization challenges are expected to arise regarding food security, health and infrastructure, to name but a few [72–76]. These continued and heightened concerns regarding the implications of the rapid pace of shifting populations in low- and middle-income countries ensure a continued demand for high-resolution gridded population maps in these regions of the world. This continued demand reinforces why understanding the drivers of the spatial distribution of populations to improve population mapping is important. Moreover, an improved understanding of the fundamental drivers of population distributions and their spatial variations is of value for modelling future growth and designing strategies around such models.

Our results show that variables related to built/urban areas and to climatic/environmental covariates were the most important for predicting population density and were the only covariate classes that were significantly more important than other variable classes, regardless of the scale of analysis. This study begins to quantify commonly held concepts regarding the drivers and correlates of human population distributions, e.g. urban areas are associated with denser populations. Having quantified these patterns globally and regionally allows future work on the more unique aspects of location-specific distributional relationships of populations to be placed within the context of these larger-scale findings, and to help relate observed and past population distributions to historical and cultural contexts and the presence or absence of resources/hazards.

The finding that built area-related covariates were the most important in predicting population density should not be a surprise and it aligns with expectations that an estimated 54% of the world's population live in urbanized areas [15]. There are numerous examples where population density was an important predictor of urban area extent [77–80]. This study shows that this relationship goes in the other direction as well with built area extent being important in predicting population density. However, caution should be used when using the newer urban/settlement feature datasets such as global human settlement layer and global urban footprint. While they are improvements on the thematically classified ‘urban’, making use of spectrally and spatially refined optical and radar-based data, they are known to be most accurate in dense urbanized areas [64,65], leading to population model biases in less densely populated or rural contexts by virtue of the settlements being missed in the input covariates [26].

We were surprised how important the climatic/environmental covariate category was in predicting population density. While the category was not broken up for subsequent testing, by examining the covariate importance plots of individual countries we believe that this importance was largely driven by elevation covariates, including derived slope. Previous studies have shown that population is prevalent in the lower elevations of resource-rich coastal zones, deltas and river valleys [81–83] and it is simply easier to build on relatively shallow–moderate slopes than on steep slopes. There is also precedence for transportation and elevation covariates being predictive of urban or built land cover, corroborated by our finding that transportation networks and climatic/environmental covariate classes were consistently important predictors of population density [27,84,85]. Water-related covariates being consistently less important than crop or natural vegetation landcover covariates (figure 3) could be a result of the resource/hazard relationship [86] that populations have with waterbodies, which of course is highly context dependent.

Differing data quality of input covariates to the models analysed here should be kept in mind when interpreting these results as they directly affect the observed importance, or non-importance, of the covariates. For instance, the significant difference seen between C. America and the Caribbean and Africa and between C. America and the Caribbean and S.E. Asia within the rivers/waterbodies/waterways covariate class (table 4) is most likely to be due to the different thematic land cover sources used for those regions. While all landcover data used were adjusted to a standard thematic framework and resampled to 100 m [27], the majority of the Africa models used the 300 m resolution Globcover data whereas the S.E. Asia and the C. America and the Caribbean data were based upon the commercial, 30 m resolution, Geocover data [28]. While C. America and the Caribbean and S.E. Asia both used the Geocover dataset, they also sourced OpenStreetMap [55] for data pertaining to river features. OpenStreetMap varies widely as to completeness, coverage and data quality [87,88]. So, we would speculate that the observed significant differences were not likely to be indicative of actual differences in how the population relates to water features between those regions, but are the result of different data sources for the built area-related covariates being used (table 2). Similar differing data quality or completeness issues are likely to be at the source of the significant differences between regions seen for the residential LU variable, which is entirely based on OpenStreetMap data [55].

These findings are valid only for a specific spatial resolution and modelling scale that may or may not maintain the same structures and relationships at a finer scale, as is typically the case with the modifiable areal unit problem (MAUP) [89]. All covariates are affected to some degree because they are all resampled to 100 m and are further aggregated by some summary measure at the administrative unit level prior to input in the RF from which our covariate importance metrics are derived [26]. Variations in data quality of the census-based population counts and the differing number of administrative units used in each region's countries modelled can partly explain the variance in importances within variable classes between regions. This follows the scale effect of the MAUP, which states that as the number of areal units is decreased there is a decrease in the variability of the observations corresponding to the areal units [89]. The potential of the coarseness of the polygonal census units to have an effect on this variability is less clear, but is likely to have an effect similar to the MAUP zonation effect [89]. So, while we observed very consistent patterns of importance between classes of variables and population density, this is based upon country-level averages of importance derived from a country-specific level of sub-national units and then analysed at the country level across all countries and between and within regional groupings of countries. Were we to change the groupings, e.g. change the level of sub-national units from which a country-level RF is constructed, then, following the MAUP, the results would be likely to change. However, given that no significant differences in importance for any covariate class between countries within a given region were found, it would appear that the regional groupings maximized internal homogeneity, better facilitating inter-regional testing for differences.

There are inferential limits to using the RF model to identify/approximate the structure of covariate class relationships to population density. Unlike multiple linear regressions or single regression trees where coefficients and confidence intervals can be quantified, the numerous trees in an RF preclude the tracing of the regression from input to prediction [37]. The strength of an RF to capture nonlinear relationships of covariates and their complex interactions, through its numerous trees, does not make for simple interpretations of the underlying mechanisms of the modelled phenomenon, in this case the driver and correlates of population distribution [37]. Covariate importance within an RF is also complex because of those same nonlinear relationships and interactions and results in a covariate's importance within an RF being highly conditional on all other covariates present, with similar results not guaranteed in other models, even for the same country [38].

Another consideration when evaluating the importance of covariate classes and their relationships to population density is the varying temporality of the covariate datasets, which may not match the date of the input census data. Therefore, the modelled relationships are imperfect to begin with, as it is impossible to have complete temporal agreement between all input datasets because of well-known availability constraints. Furthermore, the quality of census data varies from country to country as well as from census to census, with completeness and spatial resolution of the administrative units being variable.

Further investigating these covariates in relation to population density could involve utilizing a different modelling framework that would allow for more inferential power as to the structure and nature of the relationships between these covariates and population density. Additionally, focusing our study on specific covariate classes, such as the urban-/suburban-related variable classes, by sourcing novel and forthcoming datasets that help illuminate the heterogeneity within these areas, both internally and across different countries and regions, could increase the predictive ability of a population model regardless of the framework. As these population datasets are scaled up to global extent, the question occurs as to whether these trends persist in high-income regions and once a consistent set of covariates is used for modelling all countries.

Better mapping of potential trends regarding drought [90], water distribution [91], crop distribution [92] and forest distribution [93] continue to improve and refine our spatial awareness of resource distribution, change and environmental patterns, globally. The relationships between population distribution and various ancillary datasets outlined in this paper provide relevant information for future work examining how populations may react to a continually changing landscape. In addition, potential exists to integrate such temporally dynamic datasets into gridded population models for better informing population distribution, not only over space but also over time [94]. However, this study is simply a cross section of covariate relationships to population density; a key question is whether these relationships remain static or are dynamic through time and the answer to that question is of great importance to population growth models, and other population-related fields, looking backwards and forwards through time.

## Supplementary Material

Sampled Countries' Administrative Unit Overview Map

## Supplementary Material

Compressed (.7z) Extracted Data and Analysis Scripts

## Data accessibility

All original extracted data and code used for analysis has been provided in the electronic supplementary material. The large size of the original model objects precluded their inclusion in the electronic supplementary material. Requests for the original model objects should be sent to the corresponding author.
