# Supplementary figures and images for: Examining the correlates and drivers of human population distributions across low- and middle-income countries
Source: J R Soc Interface. 2017 Dec 13;14(137):20170401. doi: 10.1098/rsif.2017.0401 (PMC5746564; doi:10.1098/rsif.2017.0401)

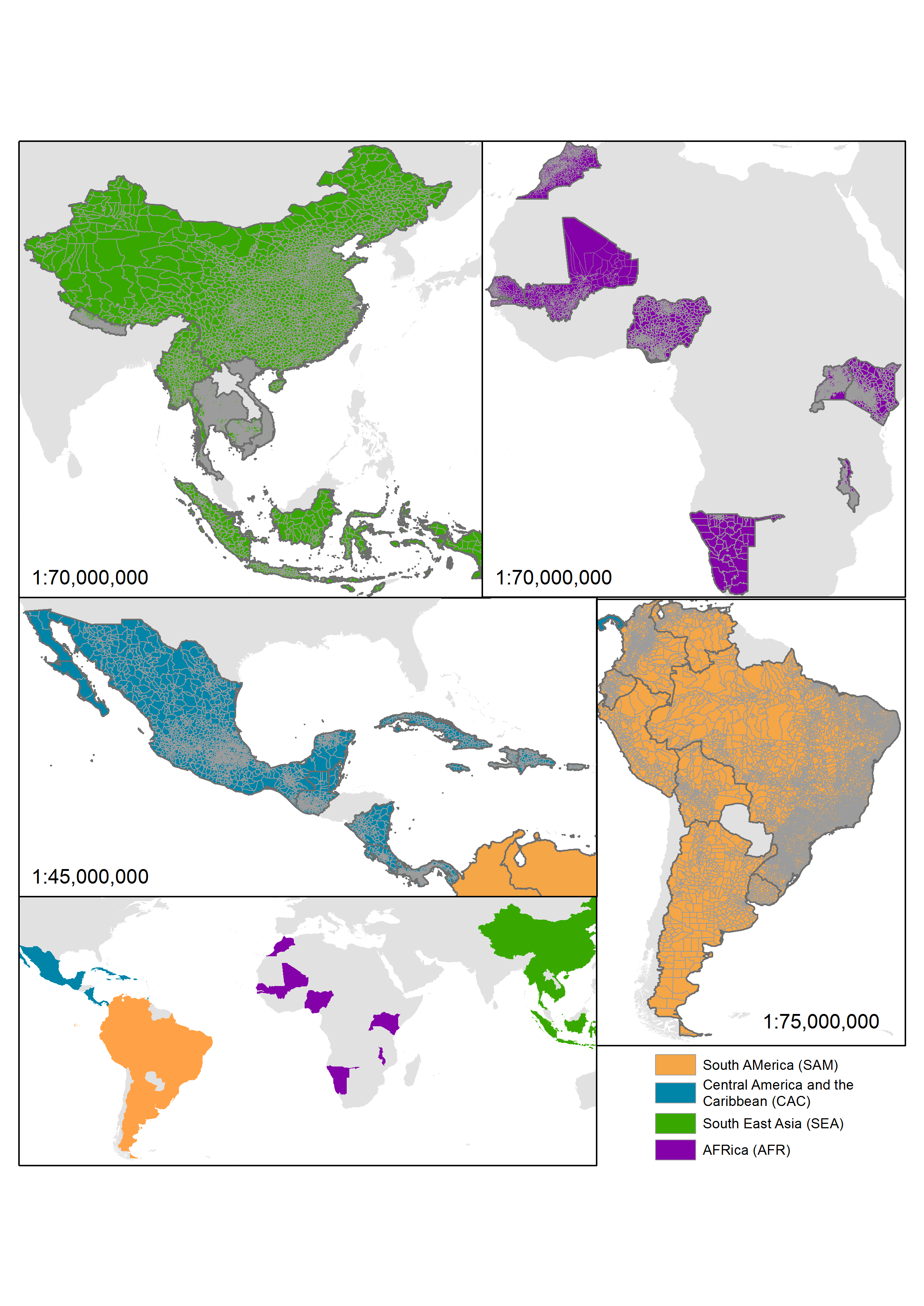

Supplement: Sampled Countries' Administrative Unit Overview Map [file rsif20170401supp1.tif]
